# Participation of Women and Older Participants in Randomized Clinical Trials of Lipid-Lowering Therapies: A Systematic Review

**DOI:** 10.1001/jamanetworkopen.2020.5202

**Published:** 2020-05-21

**Authors:** Safi U. Khan, Muhammad Zia Khan, Charumathi Raghu Subramanian, Haris Riaz, Muhammad U. Khan, Ahmad Naeem Lone, Muhammad Shahzeb Khan, Eve-Marie Benson, Mohamad Alkhouli, Michael J. Blaha, Roger S. Blumenthal, Martha Gulati, Erin D. Michos

**Affiliations:** 1 Department of Medicine, West Virginia University, Morgantown; 2 Department of Medicine, Washington Hospital Health Care System, Fremont, California; 3 Department of Cardiovascular Medicine, Cleveland Clinic, Cleveland, Ohio; 4 Department of Medicine, John H. Stroger Cook County Hospital, Chicago, Illinois; 5 Johns Hopkins School of Medicine, Ciccarone Center for the Prevention of Cardiovascular Disease, Baltimore, Maryland; 6 Department of Cardiovascular Medicine, Mayo Clinic, Rochester, Minnesota; 7 Division of Cardiology, University of Arizona College of Medicine, Phoenix

## Abstract

**Importance:**

Randomized clinical trials (RCTs) of lipid-lowering therapies form the evidence base for national and international guidelines. However, concerns exist that women and older patients are underrepresented in RCTs.

**Objective:**

To determine the trends of representation of women and older patients (≥65 years) in RCTs of lipid-lowering therapies from 1990 to 2018.

**Data Sources:**

The electronic databases of MEDLINE and ClinicalTrials.gov were searched from January 1990 through December 2018.

**Study Selection:**

RCTs of lipid-lowering therapies with sample sizes of at least 1000 patients and follow-up periods of at least 1 year were included.

**Data Extraction and Synthesis:**

Two independent investigators abstracted the data on a standard data collection form.

**Main Outcomes and Measures:**

Patterns of representation of women and older adults were examined overall in lipid-lowering RCTs and according to RCT-level specific characteristics. The participation-to-prevalence ratio (PPR) metric was used to estimate the representation of women compared with their share of disease burden.

**Results:**

A total of 60 RCTs with 485 409 participants were included. The median (interquartile range) number of participants per trial was 5264 (1062-27 564). Overall, representation of women was 28.5% (95% CI, 24.4%-32.4%). There was an increase in the enrollment of women from the period 1990 to 1994 (19.5%; 95% CI, 18.4%-20.5%) to the period 2015 to 2018 (33.6%; 95% CI, 33.4%-33.8%) (*P* for trend = .01). Among common limiting factors were inclusion of only postmenopausal women or surgically sterile women (28.3%; 95% CI, 18.5%-40.7%) or exclusion of pregnant (23.3%; 95% CI, 14.4%-35.4%) and lactating (16.6%; 95% CI, 9.3%-28.1%) women. Women were underrepresented compared with their disease burden in lipid RCTs of diabetes (PPR, 0.74), heart failure (PPR, 0.27), stable coronary heart disease (PPR, 0.48), and acute coronary syndrome (PPR, 0.51). Only 23 RCTs with 263 628 participants reported the proportion of older participants. Overall representation of older participants was 46.7% (95% CI, 46.5%-46.9%), which numerically increased from 31.6% (95% CI, 30.8%-32.3%) in the period 1995 to 1998 to 46.2% (95% CI, 46.0%-46.5%) in the period 2015 to 2018 (*P* for trend = .43). A total of 53.0% (95% CI, 41.8%-65.3%) and 36.6% (95% CI, 25.6% to 49.3%) trials reported outcomes according to sex and older participants, respectively, which did not improve over time.

**Conclusions and Relevance:**

In this systematic review of RCTs of lipid-lowering therapies, the enrollment of women and older participants increased over time, but women and older participants remained consistently underrepresented. This limits the evidence base for efficacy and safety in these subgroups.

## Key Points

**Question:**

Are women and older participants adequately represented in randomized clinical trials of lipid-lowering therapy?

**Findings:**

In this systematic review of 60 trials with 485 409 participants that were conducted between 1990 and 2018, there was a modest increase in the enrollment of women (from 19.5% to 33.6%) and older participants (from 31.6% to 46.2%); however, overall representation was low (28.5% women and 46.7% older adults). Women were underrepresented in trials compared with their relative disease burden.

**Meaning:**

The results of this study suggest that despite ongoing efforts to increase the representation of women and older participants, these subgroups remained consistently underrepresented in randomized clinical trials of lipid-lowering therapies, limiting the evidence base.

## Teaser

This systematic review and meta-analysis determines the trends of representation of women and older patients (≥65 years) in randomized clinical trials of lipid-lowering therapies from 1990 to 2018.

## Introduction

Women and older patients carry significant burden of atherosclerotic cardiovascular disease.^1,2,3,4,5^ However, concerns exist that despite the high prevalence of cardiovascular morbidity among these subgroups, they are underrepresented in clinical trials.^5,6^ Randomized clinical trials (RCTs) are considered the criterion standard for evidence-based medicine; thus, they shape guideline recommendations for patients and play a critical role in the prevention and treatment of cardiovascular disease. Given that the efficacy and toxicity of a drug are potentially influenced by several factors, including sex hormones and age-related issues of absorption and metabolism, underrepresentation of women and older adults in RCTs can undermine the generalizability of the findings to these subsets of the population.

In 1986, the National Institutes of Health advisory committee recommended inclusion of women to grant applicants.^7^ Over the decades, US Food and Drug Administration (FDA) recommendations have evolved regarding reporting of sex and other demographic variables in RCTs. In recent years, there has been an ongoing effort in industry-funded trials to recruit women and older patients.^8,9^

Lipid-lowering therapies are the cornerstone for the prevention and management of atherosclerotic cardiovascular disease, used for both secondary prevention and for higher-risk primary prevention.^10,11,12,13^ However, limited data exist to demonstrate how successful these efforts have been in improving the representation of these subgroups in large-outcome RCTs of lipid-lowering drugs. To fill this knowledge gap, we conducted a systematic review of all published large RCTs of lipid-lowering therapies from 1990 to 2018 with the goals of determining patterns of enrollment of women and older adults overall and according to RCT-level specific characteristics, assessing temporal trends in these demographic groups, and comparing results with epidemiologic studies.

## Methods

This systematic review was performed in accordance with Preferred Reporting Items for Systematic Reviews and Meta-analyses (PRISMA) reporting guideline.^14^

### Data Sources and Searches

Two of us (M.U.K. and A.N.L.) performed the literature search, using databases of MEDLINE/PubMed and ClinicalTrials.gov from January 1990 to December 2018. A broad search strategy was used with relevant search terms, as follows: *lipid*, *statin*, *proprotein convertase subtilisin/kexin type 9*, *ezetimibe*, *bile acid sequestrants*, *fibrates*, *niacin*, *diet*, *omega 3 fatty acids*, and *LDL-C* (eTable 1 in the Supplement). We also searched meta-analyses conducted on RCTs of lipid-lowering therapies for additional information.^10,11,15,16^ After removing duplicates, 2 of us (M.U.K. and A.N.L.) reviewed the records at the title and abstract level, followed by full-text screening based on predetermined study selection criteria.

### Study Selection

The prespecified inclusion criteria were as follows: (1) RCTs of lipid-lowering therapies (ie, statins, proprotein convertase subtilisin/kexin type 9 inhibitors, ezetimibe, bile acid sequestrants, fibrates, niacin, and omega-3 polyunsaturated fatty acids); (2) sample size of at least 1000 participants and follow-up duration of at least 1 year; and (3) English language. We selected large RCTs with follow-up periods of at least 1 year because large RCTs with longer follow-up are considered to provide more accurate information.^15,16^ We included RCTs of both primary and secondary prevention populations. We excluded RCTs performed among patients younger than 18 years, those reporting secondary, interim, or post hoc analyses, and RCTs of cholesteryl ester transfer protein inhibitors, given that these drugs are not approved for management of dyslipidemia.

### Data Extraction

Two of us (C.R.S. and A.N.L.) abstracted the data using prespecified data collection forms, appraised the accuracy of the data, and resolved any discrepancies by consensus after discussion with a third investigator (S.U.K.). The following information was abstracted from RCTs, as follows: title, year of publication, journal, lipid-lowering drug class, setting (ie, primary vs secondary prevention), target population or indication, total sample size, proportion of women, mean or median age, proportion of older (ie, ≥65 years) patients, number of countries involved, funding sources, proportion reporting results based on sex and age, and inclusion and exclusion criteria that would limit the recruitment of women. For missing information, ClinicalTrials.gov was reviewed for additional details.

Given that the pharmacologic interventions in the RCTs influence the biologic response, we used the term sex to refer to women.^17^ In most RCTs, sex was investigator reported.^17,18^ We categorized an RCT as primary prevention if it clearly reported primary prevention in the methods or if less than 50% of participants had atherosclerotic cardiovascular disease.^19^ We categorized RCTs according to therapy, setting, target population or indication, location, and funding sources. Consistent with earlier reports^6^ and ClinicalTrials.gov designations, funding source was classified as industry; government; nonprofit or nonfederal organizations, including university or educational institutions; and collaborative trials between nonprofit organizations and industries. We divided the locations into exclusively North America (United States, Canada, and Mexico), exclusively Western Europe (Austria, Belgium, Bermuda, Denmark, Finland, France, Germany, Greece, Iceland, Ireland, Italy, Luxembourg, the Netherlands, Norway, Portugal, Spain, Sweden, Switzerland, and United Kingdom), the rest of the world (exclusively outside North America and Western Europe), and mixed or multiregional.

### Outcome Measures

We had 4 outcomes of interest. They were prevalence of women and older participants in RCTs, temporal trends in the participation of women and older adults, representation of women in RCTs relative to their disease burden, and trends in RCTs reporting outcomes based on sex and age.

### Statistical Analysis

RCTs were placed in 4-year groups based on publication year, except for the first 5 years, which were grouped together. Continuous variables were reported as means with SDs or medians with interquartile ranges (IQRs), and categorical variables were expressed as counts with percentages. Categorical variables were compared using χ^2^ testing. Two-sided hypothesis testing was performed, with α < .05 as the level of statistical significance.

The proportion of women and older participants were trended against year of publication using simple linear regression models with a significance threshold set at 5%. To examine the representation of women in RCTs relative to their representation in populations affected by disease, we used the previously used metric of participation-to-prevalence ratio (PPR),^5,20,21^ which is derived by dividing the percentage of women among trial participants by the percentage of women in the disease population. The denominators were obtained from studies reporting the most recent or epidemiologic population-based data representing global disease burden (eTable 2 in the Supplement). If global data were not provided in a single study, we weighted the regional data (derived by multiplying the mean of each study by the weighted number based on the study’s size relative to the total studies included). The interpretation of PPR values was based on thresholds used in prior studies,^5,20,21^ ie, less than 0.8 indicates underrepresentation; greater than 1.2, overrepresentation; and close to 1.0, adequate representation. For example, if the prevalence of women in the disease population is 60% and RCTs in this disease enrolled 40% women, the PPR would be 40% / 60% = 0.66, indicating underrepresentation. We selected disease populations of acute coronary syndrome (ACS), stable coronary heart disease (CHD), heart failure (HF), diabetes, and hypercholesteremia. To estimate the prevalence of women among the disease population, we divided the prevalence or the incidence of that disease among women by the total prevalence or incidence of that disease^5^ (eTable 2 in the Supplement). The PPR was not calculated for older patients because of the small number of RCTs that reported participation information. Analyses were performed with SPSS statistical software version 24 (IBM Corp) and Excel version 16 (Microsoft Corp).

## Results

**Figure 1.**
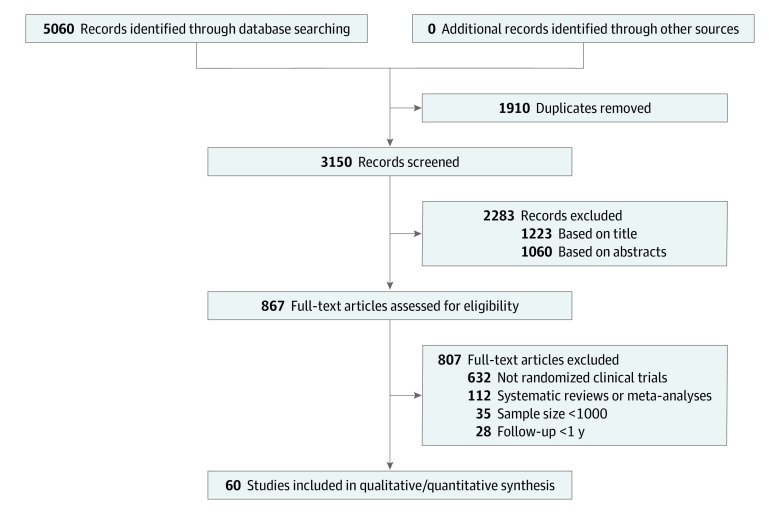
Flow Diagram of Selection Process

Of 3150 records screened after removal of duplicates, 60 RCTs with 485 409 participants were included (Figure 1). The list of included RCTs is given in eTable 3 in the Supplement. The median (IQR) number of participants per RCT was 5264 (1062-27 564). A total of 32 RCTs (53.3%) of statins with 196 951 participants, 12 RCTs (20.0%) of omega-3 polyunsaturated fatty acids with 126 907 participants, 6 RCTs (10.0%) of proprotein convertase subtilisin/kexin type 9 inhibitors with 80 675 participants, 3 RCTs (5.0%) of ezetimibe with 29 287 participants, 5 RCTs (8.3%) of fibrates with 22 502 participants, and 2 RCTs (3.3%) of niacin therapy with 29 087 participants were included (Table). A total of 32 RCTs (52.2%) with 260 915 participants were of secondary prevention, and 28 RCTs (47.8%) with 224 494 participants were of primary prevention. Participants with ACS were the most commonly studied population (16 RCTs [27.7%] with 113 339 patients), followed by stable CHD (15 RCTs [25.0%] with 142 565 patients), and hypercholesterolemia (7 RCTs [11.7%] with 70 175 patients). Overall, 21 RCTs (35.0%) with 142 034 participants were conducted in Western Europe, 20 RCTs (33.3%) with 190 358 participants were multiregional, and 13 RCTs (21.7%) with 93 317 participants were conducted in North America. More than half of the RCTs (35 [58.3%] with 267 631 participants) were industry funded, 5 RCTs (8.3%) with 22 071 participants were funded by universities or other nonfederal and nonprofit organizations, and 3 RCTs (5.0%) with 35 592 participants were government funded.

**Table.** Trends in Age and Representation of Women in Lipid-Lowering Therapy Randomized Clinical Trials Over Time

Characteristic	No. (%) by publication year							*P* value
	1990-1994	1995-1998	1999-2002	2003-2006	2007-2010	2011-2014	2015-2018	
Trials, No.	2	5	11	13	12	6	11	NA
Total participants, No.	5506	27 724	65 842	71 258	86 424	67 601	161 054	NA
Participants per trial, median (IQR)	2753 (NA)	6595 (2755-7809)	3090 (1677-10 355)	4497 (2426-9341)	4924 (3042-11 610)	10 887 (4006-15 820)	15 480 (8179-18 924)	.01
Weighted age, mean (SD)	56.5 (2.1)	59.0 (2.7)	63.4 (5.0)	60.3 (3.6)	65.4 (3.7)	65.0 (4.5)	62.3 (2.8)	<.001
Trials reporting age ≥65 y	0	2 (40.0)	4 (36.4)	3 (23.1)	3 (25.0)	4 (66.7)	7 (63.6)	.35
Participants with age ≥65 y	0	4930 (31.6)	19 082 (50.6)	6055 (37)	12 479 (47.8)	28 102 (51.9)	52 589 (46.2)	.43
Women	1074 (19.5)	3200 (11.5)	17 339 (26.3)	21 943 (30.7)	31 379 (36.3)	22 878 (33.8)	5424 (33.6)	.01
Therapy								
Statins	2 (100)	5 (100)	7 (63.6)	12 (92.3)	5 (41.7)	0	1 (9.1)	<.001
Ezetimibe	0	0	0	0	1 (8.3)	1 (16.7)	1 (9.1)	
PCSK9 inhibitors	0	0	0	0	0	0	6 (54.5)	
Fibrates	0	0	3 (27.3)	1 (7.7)	1 (8.3)	0	0	
Niacin	0	0	0	0	0	2 (33.3)	0	
Omega-3 fatty acids	0	0	1 (9.1)	0	5 (41.7)	3 (50.0)	3 (27.3)	
Indication or baseline population								
Aortic stenosis	0	0	0	0	1 (8.3)	0	0	.31
Chronic kidney disease	0	0	0	2 (15.4)	1 (8.3)	1 (16.7)	0	
Diabetes	0	0	0	3 (23.1)	1 (8.3)	1 (16.7)	1 (9.1)	
Hypercholesterolemia	0	2 (40.0)	1 (9.1)	1 (7.7)	2 (16.7)	0	1 (9.1)	
Hypercholesterolemia with risk factors for ASCVD	1 (50.0)	0	1 (9.1)	0	0	1 (16.7)	2 (18.2)	
Risk factors for ASCVD without hypercholesteremia	0	0	0	1 (7.7)	0	1 (16.7)	1 (9.1)	
Acute coronary syndrome	0	3 (60.0)	5 (45.5)	3 (23.1)	3 (25)	0	2 (18.2)	
Stable coronary heart disease	1 (50.0)	0	4 (36.4)	3 (23.1)	1 (8.3)	2 (33.3)	4 (36.4)	
Heart failure	0	0	0	0	3 (25)	0	0	
Setting								
Primary prevention	1 (50.0)	2 (40.0)	2 (18)	7 (53.8)	7 (58.3)	4 (66.7)	5 (45.5)	.47
Secondary prevention	1 (50.0)	3 (60.0)	9 (81)	6 (46.2)	5 (41.7)	2 (33.3)	6	
Location								
North America	0	3 (60.0)	2 (18.2)	3 (23.1)	1 (8.3)	2 (33.3)	2 (18.2)	.25
Western Europe	1 (50.0)	1 (20.0)	5 (5.5)	4 (30.8)	7 (58.3)	2 (33.3)	1 (9.1)	
Multiregional	1 (50.0)	0	2 (18.2)	4 (30.8)	3 (25.0)	2 (33.3)	8 (72.7)	
Rest of the world	0	1 (20.0)	2 (18.2)	2 (15.4)	1 (8.3)	0	0	
Funding								
Industry	2 (100)	1 (20.0)	6 (54.5)	9 (69.2)	8 (66.7)	1 (16.7)	8 (72.7)	.13
Government	0	0	0	0	1 (8.3)	1 (16.7)	1 (9.1)	
University/organization	0	0	3 (27.3)	1 (7.7)	1 (8.3)	0	0	
Other/combined	0	4 (80.0)	2 (18.2)	3 (23.1)	2 (16.7)	4 (66.7)	2 (18.2)	

Abbreviations: ASCVD, atherosclerotic cardiovascular disease; NA, not applicable; PCSK9, proprotein convertase subtilisin/kexin type 9.

### Prevalence of Women and Older Participants

Overall representation of women was 28.5% (95% CI, 24.4%-32.4%). The representation of women varied from 17.0% (95% CI, 16.5%-17.4%) in niacin RCTs to 42.7% (95% CI, 42.4%-42.9%) in RCTs of omega-3 polyunsaturated fatty acids (*P* = .04) (eTable 4 in the Supplement). The enrollment of women was higher in primary prevention trials (42.2%; 95% CI, 41.9%-42.3%) vs secondary prevention trials (22.0%; 95% CI, 21.8%-22.1%) (*P* = .02). There were no significant differences based on target population or indication, location, or funding sources of trials.

A total of 28 trials (46.7%) highlighted women in their selection criteria. Among women, the most common inclusion and exclusion criteria were postmenopausal women or surgically sterile women, who constituted 28.3% (95% CI, 18.5%-40.7%) of study participants, and pregnant women, who constituted 23.3% (95% CI, 14.4%-35.4%) of study participants (eTable 5 in the Supplement). Other exclusion criteria were based on age thresholds or factors such as lactation (with 16.6% [95% CI, 9.3%-28.1%] of study participants).

Mean (SD) age of participants was highest in RCTs of patients with HF (69.3 [3.2] years) compared with trials of patients with stable CHD (62.6 [2.5] years) and ACS (61.8 [2.9] years) (*P* = .03) (eTable 6 in the Supplement). A total of 23 RCTs (38.3%) with 263 628 participants reported the proportion of older participants (eTable 7 in the Supplement). Overall representation of older participants was 46.7% (95% CI, 46.5%-46.9%). There were no significant differences in proportion of older participants based on therapy, setting, location, or funding sources. A total of 28 RCTs (46.7%) set age thresholds for inclusion criteria. The most common limiting age criteria were being younger than 75 years (20.0%; 95% CI, 11.8%-31.7%) followed by being younger than 80 years (15.0%; 95% CI, 8.0%-26.1%) (eTable 5 in the Supplement).

### Temporal Trends in Women and Older Participants

**Figure 2.**
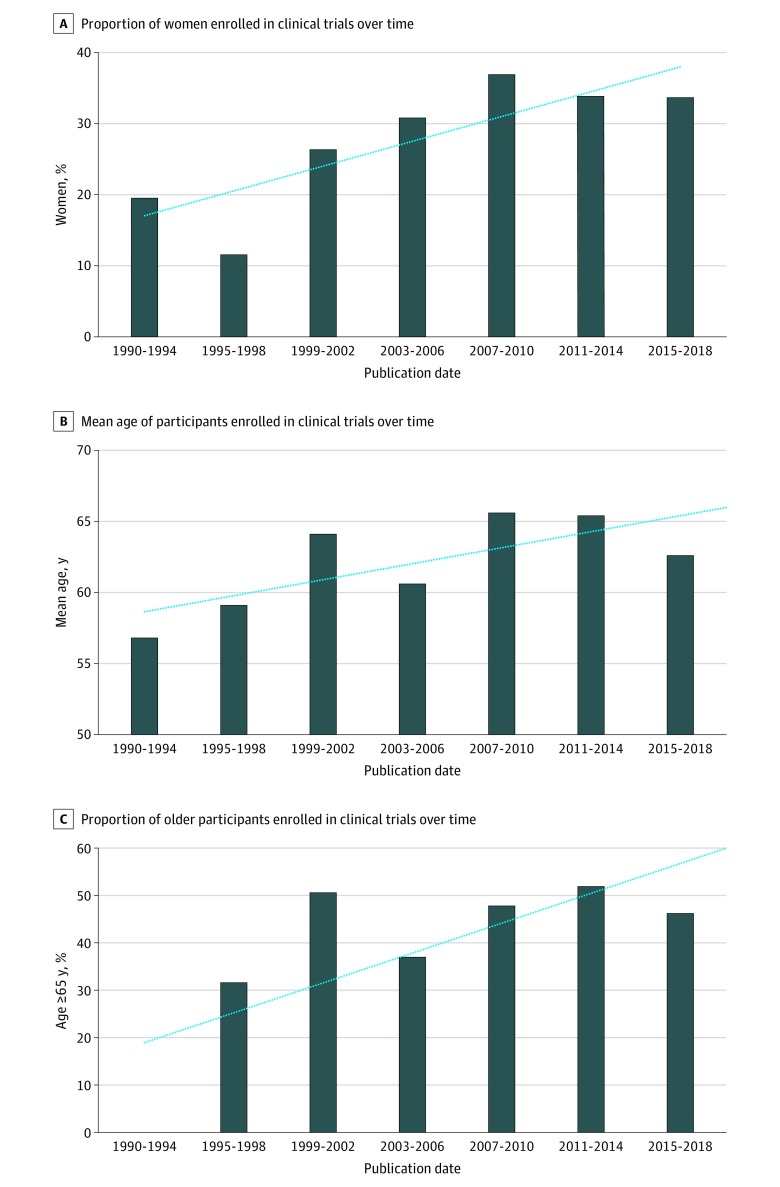
Patients Enrolled in Lipid-Lowering Therapy Randomized Clinical Trials Over Time Blue dotted lines indicate linear trend.

There was a modest increase in the enrollment of women between 1990 to 1994 (19.5%; 95% CI, 18.4%-20.5%) and 2015 to 2018 (33.6%; 95% CI, 33.4%-33.8%) (*P* for trend = .01) (Figure 2A). Mean (SD) age of participants increased between RCTs in 1990 to 1994 (56.8 [2.5] years) and in 2015 to 2018 (62.6 [2.8] years) (*P *for trend < .001) (Figure 2B). The representation of older participants numerically increased from 31.6% (95% CI, 30.8%-32.3%) in 1995 to 1998 to 46.2% (95% CI, 46.0%-46.5%) in 2015 to 2018 (*P *for trend = .43) (Figure 2C).

### Representation of Women in Trials Compared With Their Disease Burden

**Figure 3.**
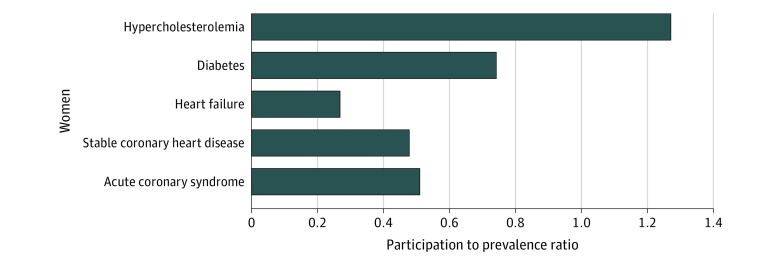
Participation-to-Prevalence Ratio of Women in Lipid-Lowering Therapy Randomized Clinical Trials, Prevalence-Corrected Estimate The ratio of the percentage of women among trial participants to the percentage of women among the disease population is the participation-to-prevalence ratio. A participation-to-prevalence ratio of 1.0 indicates that the sex composition of the randomized clinical trial was equal to that of the disease population. A participation-to-prevalence ratio between 0.8 and 1.2 indicates that proportion of women in the trial was similar to the proportion of women in the disease population.

Women were underrepresented compared with their share of the disease population in trials of diabetes (PPR, 0.74), HF (PPR, 0.27), stable CHD (PPR, 0.48), and ACS (PPR, 0.51) (Figure 3). However, women in trials of hypercholesterolemia were overrepresented compared with their proportion in disease population (PPR, 1.27).

### Trends in Trials Reporting Outcomes Based on Sex and Age

**Figure 4.**
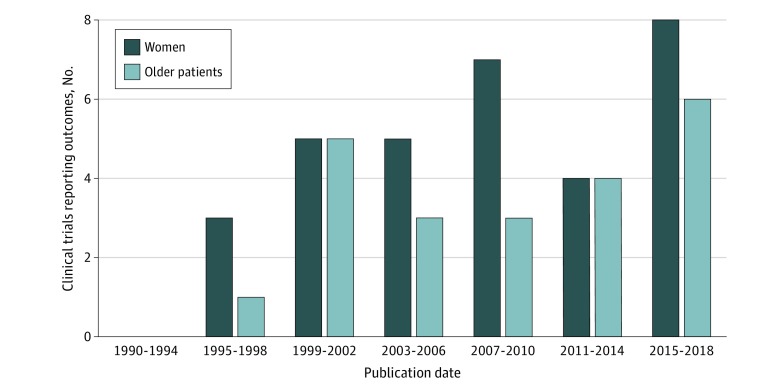
Number of Lipid-Lowering Therapy Randomized Clinical Trials Reporting Clinical Outcomes Based on Subgroups of Women and Older Participants

A total of 53.0% (95% CI, 41.8% to 65.3%) RCTs reported outcomes according to sex, which did not increase over time (*P *for trend = .42) (Figure 4; eTable 8 in the Supplement). Among these, statin RCTs had the highest (53.0% [95% CI, 36.4%-69.1%]) reporting of outcomes according to sex. A total of 36.6% (95% CI, 25.6%-49.3%) of RCTs reported outcomes in older participants, which did not increase over time (*P* for trend = .20). Of these, statin trials had the highest (28.1%; 95% CI, 15.5%-45.3%) reporting of outcomes according to age.

## Discussion

In this systematic review, we found that while there was a modest increase in the enrollment of women and older patients over time, these demographic subgroups generally remained underrepresented in trials of lipid-lowering therapy. This pattern was consistent across primary region of enrollment and type of drug class. Enrollment of women varied by trial funding sources, mean age of participants varied according to baseline indication of therapy, and enrollment of older participants varied according to therapy. Reporting of outcomes based on sex and age was poor, with nearly half of the included RCTs reporting estimates for women and less than half for older participants.

We compared our results with epidemiologic studies and surveys to assess discrepancies among recruitment of these key demographic groups in RCTs with the disease prevalence among them. Except for RCTs studying hypercholesterolemia, women were underrepresented in lipid trials of diabetes and CVD compared with their relative share of the disease burden in the population. In terms of age, approximately 15% of the US population is aged 65 years older, of whom 24% are reported to have CHD and 25% are reported to have diabetes.^3,6,22^ Based on epidemiologic surveys, the mean age of patients with HF is approximately 74 years.^6^ The mean ages of participants with CHD, diabetes, and HF in our study were 63, 62 and 69 years, respectively. However, with respect to disease, older participants’ representation in trials appeared to be at a similar level or higher than their disease prevalence. For instance, the prevalence of CHD and diabetes was nearly 24% and 25%, respectively, in patients aged 65 years or older,^3,23^ whereas enrollment of older participants in RCTs for CHD and diabetes were 48.1% and 46.0% respectively. However, the information in our study was derived from the included RCTs that provided this data (approximately 32%), which might overestimate the prevalence of older participants.

The potential explanations for these pattern are as follows: first, as illustrated, disproportionate inclusion and exclusion criteria of RCTs favoring men over women^5,9^; second, limited screening of women for enrollment either because of possible implicit biases or other social or medical reasons that make their participation difficult^5,9,24^; third, lack of willingness to participate in clinical trials; fourth, compounded issues related to underenrollment of patients from minority groups because of language barriers, disparities in socioeconomic position, and unique cultural practices that limit their participation^25,26^; and fifth, exclusion criteria based on arbitrary age thresholds or factors that could limit participation of older participants, such as concerns about competing causes of death from cancer or comorbidities, decreased mobility or worsening cognitive function that might limit participation in RCTs, use of medications affecting the study protocol, and visual and hearing impairments.^27^ While the FDA guidelines in 1989 stated that there is no clear basis to exclude participants based on age, the National Institutes of Health Revitalization Act of 1993 was limited to women and patients from minority groups and did not include provisions for older patients.^28^

Moving forward, to improve the representation of women and older participants, investigators should strictly follow the 2012 FDA position statement.^29,30^ Using a site-based approach, intensive and innovative strategies should be adapted involving investigators, sponsors, and community members to enhance recruitment of these subsets of the population, considering cultural sensitivity, simplifying consent forms, and potentially including incentives, such as childcare, transportation, or access to medical care.^6,26^ Following similar footsteps, RCTs have been shown to improve the representation of demographic subgroups.^31^

### Limitations

Our study has certain limitations. This is an RCT-level systematic review, and we did not have access to individual participant data. However, other than the inherent challenges in accruing the data of lipid-lowering RCTs that span decades, we do not believe that would have changed the results of this analysis. We selected large RCTs with at least 1 year follow-up, given that large studies with extended follow-ups are more likely to provide evidence that would shape guideline recommendations for patients^6,16^; however, a degree of selection bias by ignoring small trials is inevitable. We did not review the effectiveness of the intervention based on demographic subgroups given the substantial heterogeneities in lipid-lowering drugs and baseline population. Moreover, because only a limited number of RCTs reported subgroup analyses, their pooled results would have been underpowered to generate any meaningful information. Given the descriptive nature of our analyses, we did not perform adjustments of the significance threshold for secondary and subgroup analyses. Therefore, these analyses should be interpreted as exploratory. Furthermore, we did not examine the recruitment trends of participants from ethnic/racial minority groups, considering that RCTs were conducted in different regions of the world and might under- or overrepresent the ethnic/racial make-up of the current study population.

## Conclusions

In this study, we reported on the representation of women and older patients in RCTs of lipid-lowering therapies. Women were underrepresented in RCTs compared with their relative disease burden in the population. While the participation of women and older participants seemed to have modestly improved with the passage of time, which is likely reflective of FDA regulations, overall the representation of these demographic groups remained disappointingly low. Clinical trials are designed to ascertain the efficacy and/or safety of a drug or device in a target population, and underenrollment limits the evidence-base for these key demographic groups. There are no legal or regulatory requirements that mandate the investigators accrue a specific percentage of participants based on sex or age. Therefore, practical steps should be undertaken to develop new strategies to achieve optimal recruitment of these subsets of the population in RCTs, and investigators should be encouraged to report results based on these subgroups to enhance generalizability of their results.

## Notes

Supplement. **eTable 1. **PubMed Search Strategy **eTable 2. **Estimation of Percentage of Women in Disease Populations **eTable 3. **List of Randomized Clinical Trials Included in Systematic Review **eTable 4. **Women Representation in Lipid-Lowering Therapy Randomized Clinical Trials **eTable 5. **Inclusion/Exclusion Criteria Focusing on Women and Older Participants in Randomized Clinical Trials **eTable 6. **Mean Age of Enrolled Patients Within Lipid-Lowering Therapy Randomized Clinical Trials **eTable 7. **Representation of Older (≥ 65 years) Participants in Lipid-Lowering Therapy Randomized Clinical Trials **eTable 8. **Trends in Randomized Clinical Trials Reporting Outcomes Based on Sex and Age **eReferences.**
