# Supplementary material for: Participation of Women and Older Participants in Randomized Clinical Trials of Lipid-Lowering Therapies: A Systematic Review
Source: JAMA Netw Open. 2020 May 21;3(5):e205202. doi: 10.1001/jamanetworkopen.2020.5202 (PMC7243092; doi:10.1001/jamanetworkopen.2020.5202)
Supplement: Supplement. — eTable 1. PubMed Search Strategy eTable 2. Estimation of Percentage of Women in Disease Populations eTable 3. List of Randomized Clinical Trials Included in Systematic Review eTable 4. Women Representation in Lipid-Lowering Therapy Randomized Clinical Trials eTable 5. Inclusion/Exclusion Criteria Focusing on Women and Older Participants in Randomized Clinical Trials eTable 6. Mean Age of Enrolled Patients Within Lipid-Lowering Therapy Randomized Clinical Trials eTable 7. Representation of Older (≥ 65 years) Participants in Lipid-Lowering Therapy Randomized Clinical Trials eTable 8. Trends in Randomized Clinical Trials Reporting Outcomes Based on Sex and Age eReferences. [file jamanetwopen-3-e205202-s001.pdf]

Supplementary Online Content

Khan SU, Khan MZ, Raghu Subramanian C, et al. Participation of women and older participants in randomized clinical trials of lipid-lowering therapies: a systematic review. *JAMA Netw Open*. 2020;3(5):e205202. doi:10.1001/jamanetworkopen.2020.5202

eTable 1. PubMed Search Strategy

eTable 2. Estimation of Percentage of Women in Disease Populations

eTable 3. List of Randomized Clinical Trials Included in Systematic Review

eTable 4. Women Representation in Lipid-Lowering Therapy Randomized Clinical Trials

eTable 5. Inclusion/Exclusion Criteria Focusing on Women and Older Participants in Randomized Clinical Trials

eTable 6. Mean Age of Enrolled Patients Within Lipid-Lowering Therapy Randomized Clinical Trials

eTable 7. Representation of Older (≥ 65 years) Participants in Lipid-Lowering Therapy Randomized Clinical Trials

eTable 8. Trends in Randomized Clinical Trials Reporting Outcomes Based on Sex and Age

eReferences.

This supplementary material has been provided by the authors to give readers additional information about their work.

© 2020 Khan SU et al. *JAMA Network Open.*

eTable 1. PubMed Search Strategy

N	Search Terms	Records
1	*Lipid AND *Lowering	4841
2	*LDL AND *Lowering	2507
3	*Cholesterol AND *Lowering	3685
4	*Statin AND *Cholesterol	4161
5	*Statin AND *LDL	3169
6	*Statin AND *Lipid	5052
7	*Ezetimibe AND *Cholesterol	475
8	*Ezetimibe AND *LDL	424
9	*Ezetimibe AND *Lipid	496
10	*Proprotein convertase subtilisin/kexin type 9 AND *Cholesterol	104
11	*Proprotein convertase subtilisin/kexin type 9 AND *LDL	98
12	*Proprotein convertase subtilisin/kexin type 9 AND *Lipid	100
13	*Bile acid sequestrants AND *Cholesterol	27
14	*Bile acid sequestrants AND *LDL	20
15	*Bile acid sequestrants AND *Lipid	25
16	*Fibrates AND *Cholesterol	951
17	*Fibrates AND *LDL	601
18	*Fibrates AND *Lipid	1223
19	*Niacin AND *Cholesterol	273
20	*Niacin AND *LDL	187
21	*Niacin AND *Lipid	345
22	*Omega 3 fatty acid AND *Cholesterol	700
23	*Omega 3 fatty acid AND *LDL	418
24	*Omega 3 fatty acid AND *Lipid	3252

© 2020 Khan SU et al. *JAMA Network Open.*

eTable 2. Estimation of Percentage of Women in Disease Populations

Disease	Region	% in disease population	Source
Acute Coronary Syndrome	USA	43	Mozaffarian D, Benjamin EJ, Go AS, et al. Heart Disease and Stroke Statistics-2016 Update: A Report From the American Heart Association. Circulation. 2016 Jan 26;133(4):e38-e360, p. 296.
	Asia	45.9	Greenslade JH, Cullen L, Parsonage W, et al. Examining the signs and symptoms experienced by individuals with suspected acute coronary syndrome in the Asia-Pacific region: a prospective observational study. *Ann Emerg Med*. 2012;60:777-785 e3
	Europe	30.5	Andre R, Bongard V, Elosua R, et al. International differences in acute coronary syndrome patients' baseline characteristics, clinical management and outcomes in Western Europe: the EURHOBOP study. *Heart*. 2014;100:1201-1207
Coronary Heart Disease	Global	48.7	Roth GA, Johnson C, Abajobir A, et al. Global, Regional, and National Burden of Cardiovascular Diseases for 10 Causes, 1990 to 2015. *J Am Coll Cardiol.* 2017;70(1):1-25
Heart Failure	USA	53	Mozaffarian D, Benjamin EJ, Go AS, et al. Heart Disease and Stroke Statistics-2016 Update: A Report From the American Heart Association. Circulation. 2016 Jan 26;133(4):e38-e360, p. e274
	China	42.5	Yu SB, Cui HY, Qin M, et al. [Characteristics of in-hospital patients with chronic heart failure in Hubei province from 2000 to 2010]. *Zhonghua Xin Xue Guan Bing Za Zhi*. 2011;39:549-552.10

© 2020 Khan SU et al. *JAMA Network Open.*

Disease	Region	% in disease population	Source
	Japan	30.2	Shiba N, Nochioka K, Miura M, Kohno H, Shimokawa H and Investigators C-. Trend of westernization of etiology and clinical characteristics of heart failure patients in Japan--first report from the CHART-2 study. *Circ J*. 2011;75:823-833.11
	Europe	32.5	Maggioni AP, Dahlstrom U, Filippatos G, Chioncel O, Leiro MC, Drozdz J, Fruhwald F, Gullestad L, Logeart D, Metra M, Parissis J, Persson H, Ponikowski P, Rauchhaus M, Voors A, Nielsen OW, Zannad F, Tavazzi L and Heart Failure Association of ESC. EURObservational Research Programme: the Heart Failure Pilot Survey (ESC-HF Pilot). *Eur J Heart Fail*. 2010;12:1076-1084.
Diabetes Mellitus	Global	48	GBD Compare | IHME Viz Hub. Vizhub.healthdata.org. https://vizhub.healthdata.org/gbd-compare/. [Accessed January 9, 2020.]
Hypercholesteremia	Global	35.8	Venkitachalam L, Wang K, Porath A, et al. Global variation in the prevalence of elevated cholesterol in outpatients with established vascular disease or 3 cardiovascular risk factors according to national indices of economic development and health system performance. *Circulation.* 2012;125(15):1858-1869.

© 2020 Khan SU et al. *JAMA Network Open.*

eTable 3. List of Randomized Clinical Trials Included in Systematic Review

Trial Name	Participants
**STATIN**	
PMSGCRP[1]	1062
4S[2]	4444
WOSCOP[3]	6595
CARE[4]	4159
POST CABG[5]	1351
AFCAPS-TexCAPS[6]	6605
LIPID[7]	9014
GISSI-P[8]	4271
MIRACL[9]	3086
ALLHAT-LLT[10]	10355
GREACE[11]	1600
HPS[12]	20536
LIPS[13]	1677
PROSPER[14]	5804
ALERT[15]	2102
ASCOT-LLA[16]	10305
A to Z[17]	4497
ALLIANCE[18]	2442
CARDS[19]	2838
PROVE-IT TIMI 22[20]	4162
TNT[21]	10001
4D[22]	1255
IDEAL[23]	8888
ASPEN[24]	2410
MEGA[25]	7832
SPARCL[26]	4731
CORONA[27]	5011
JUPITER[28]	17802
GISSI-HF[29]	4574
AURORA[30]	2773
SEARCH[31]	12064
HOPE-3[32]	12705
**EZETIMIBE**	
SEAS[33]	1873
SHARP[34]	9270
IMPROVE-IT[35]	18144
**PCSK9 INHIBITOR**	
ODYSSEY LONGTERM[36]	2341
OSLER[37]	4465

© 2020 Khan SU et al. *JAMA Network Open.*

FOURIER[38]	27564
SPIRE 1[39]	16817
SPIRE 2[39]	10564
ODYSSEY OUTCOMES[40]	18924
**FIBRATES**	
BIP[41]	3090
LEADER[42]	1568
FIELD[43]	9795
ACCORD[44]	5518
VA-HIT[45]	2531
**NIACIN**	
AIM-HIGH[46]	3414
HPS2-THRIVE[47]	25673
**OMEGA 3 FATTY ACID**	
AREDS-2[48]	4203
SU.FOL.MO3[49]	2501
JELIS[50]	18645
ALPHA OMEGA[51]	4837
OMEGA[52]	3851
R&P[53]	12505
GISSI-HF[54]	6975
ORIGIN[55]	12536
GISSI-P[56]	11324
VITAL[57]	25871
ASCEND[58]	15480
REDUCE-IT[59]	8179

© 2020 Khan SU et al. *JAMA Network Open.*

eTable 4. Women Representation in Lipid-Lowering Therapy Randomized Clinical Trials

	No. of Trials	Overall Population	Women, No. (%)	P-value
**Publication Year**				
1990-1994	2	5506	1074 (19.5)	0.01
1995-1998	5	27724	3200 (11.5)	
1999-2002	11	65842	17339 (26.3)	
2003-2006	13	71258	21943 (30.7)	
2007-2010	12	86424	31379 (36.3)	
2011-2014	6	67601	22878 (33.8)	
2015-2018	11	161054	54224 (33.6)	
**Therapy**				
Statins	32	196951	55940 (28.4)	0.04
Ezetimibe	3	29287	8609 (29.3)	
PCSK9 inhibitors	6	80675	22696 (28.1)	
Fibrates	5	22502	5634(25.0)	
Niacin	2	29087	4948 (17.0)	
Omega 3 fatty acids	12	126907	54210 (42.7)	
**Setting**				
Primary prevention	28	224494	94662 (42.2)	0.02
Secondary prevention	32	260915	57375 (22.0)	
**Target population/indication**				
Aortic stenosis	1	1873	723 (38.6)	0.08
Chronic kidney disease	4	15400	5813 (37.7)	
Diabetes mellites	6	48577	17271 (35.6)	
Hypercholesterolemia	7	70175	31875 (45.4)	
Hypercholesterolemia with risk factors for ASCVD	5	36541	19018 (52.0)	
Risk factors for ASCVD without hypercholesteremia	3	40379	17414 (43.1)	
Acute coronary syndrome	16	113339	23039 (20.3)	
Stable coronary heart disease	15	142565	33156 (23.3)	

© 2020 Khan SU et al. *JAMA Network Open.*

Heart failure	3	16560	3728 (10.6)	
**Location**				
North America	13	93317	35526 (38.1)	0.35
Western Europe	21	142034	39791 (28)	
Multiregional	20	190358	53457 (28)	
Rest of the world	6	59700	25263 (42)	
**Funding**				
Industry	35	267631	82673 (31)	0.14
Government	3	35592	17166 (48.2)	
University/organization	5	22071	5660 (26)	
Other/combined	17	160115	46538 (29)	

© 2020 Khan SU et al. *JAMA Network Open.*

eTable 5. Inclusion/Exclusion Criteria Focusing on Women and Older Participants in Randomized Clinical Trials

Inclusion/Exclusion criteria	Percentage [95% CI]
**Women**	
All women excluded	5.0% (1.7% to 13.7%)
Pregnant women excluded	23.3% (14.4% to 35.4%)
Lactating women excluded	16.6% (9.3% to 28.1%)
Only postmenopausal women or surgically sterile women included	28.3% (18.5%to 40.7%)
Only women >55 years included	3.3% (0.9% to 11.3%)
Only women >60 years included	5.0% (1.7% to 13.7%)
Only >65 years included	1.6% (0.2% to 8.8%)
**Older participants (inclusion criteria)**	
<65 years	1.6% (0.2% to 8.8%)
<70 years	3.3% (0.9% to 11.3%)
<75 years	20.0% (11.8% to 31.7%)
<79 years	1.6% (0.2% to 8.8%)
<80 years	15.0% (8.0% to 26.1%)
<82 years	1.6% (0.2% to 8.8%)
<85 years	1.6% (0.2% to 8.8%).

© 2020 Khan SU et al. *JAMA Network Open.*

eTable 6. Mean Age of Enrolled Patients Within Lipid-Lowering Therapy Randomized Clinical Trials

	No. of Trials	Total Population	Mean age (SD)	P-value
**Publication Year**				
1990-1994	2	5506	56.8 (2.5)	<0.001
1995-1998	5	27724	59.1 (2.8)	
1999-2002	11	65842	64.1 (5.1)	
2003-2006	13	71258	60.6 (3.7)	
2007-2010	12	86424	65.6 (3.6)	
2011-2014	6	67601	65.4 (4.5)	
2015-2018	11	161054	62.6 (2.8)	
**Therapy**				
Statins	32	196951	62.1 (5)	0.41
Ezetimibe	3	29287	64.4 (2.8)	
PCSK9 inhibitors	6	80675	60.9 (2.2)	
Fibrates	5	22502	63.4 (3.2)	
Niacin	2	29087	64.3 (0.8)	
Omega 3 fatty acids	12	126907	64.8 (4.1)	
**Setting**				
Primary Prevention	28	224494	63.1 (5.4)	0.66
Secondary Prevention	32	260915	62.6 (3.3)	
**Target population/indication**				
Aortic stenosis	1	1873	67.5 (NA)	0.03
Chronic kidney disease	4	15400	60.5 (7.1)	
Diabetes mellites	6	48577	62.3 (1)	
Hypercholesterolemia	7	70175	60.8 (4.2)	
Hypercholesterolemia with risk factors for ASCVD	5	36541	63.6 (7.9)	
Risk factors for ASCVD without hypercholesteremia	3	40379	68.2 (5.7)	
Acute coronary syndrome	16	113339	61.8 (2.9)	
Stable coronary heart disease	15	142565	62.6 (2.5)	
Heart failure	3	16560	69.3 (3.2)	

© 2020 Khan SU et al. *JAMA Network Open.*

**Location**				
North America	13	93317	63.3 (4.4)	0.24
Western Europe	21	142034	64.1 (4.5)	
Multiregional	20	190358	62.0 (4.6)	
Rest of the world	6	59700	60.5 (1.5)	
**Funding**				
Industry	35	267631	62.4 (4.6)	0.16
Government	3	35592	67.9 (6.1)	
University/organization	5	22071	64.6 (5.1)	
Other/combined	17	160115	62.3 (2.8)	

© 2020 Khan SU et al. *JAMA Network Open.*

eTable 7. Representation of Older (≥ 65 years) Participants in Lipid-Lowering Therapy Randomized Clinical Trials

	No. of Trials	Overall Population	Older participants, No. (%)	P-values
**Publication Year**				
1990-1994	NR	NR	NR	
1995-1998	2	15619	4930 (31.6)	0.43
1999-2002	4	37693	19082 (50.6)	
2003-2006	3	16367	6055 (37.0)	
2007-2010	3	26093	12479 (47.8)	
2011-2014	4	54128	28102 (51.9)	
2015-2018	7	113728	52589 (46.2)	
**Therapy**				
Statins	10	90633	42112 (46.5)	0.49
Ezetimibe	1	18144	7971(43.9)	
PCSK9 inhibitors	3	48829	18187 (37.2)	
Fibrates	3	17844	7079 (39.7)	
Niacin	2	29087	14323 (49.2)	
Omega 3 fatty acids	4	59091	33565 (56.8)	
**Setting**				
Primary Prevention	12	121216	61723 (50.9)	0.81
Secondary Prevention	11	142412	61514 (43.2)	
**Target population/indication**				
Aortic stenosis	0	0	0	0.14
Chronic kidney disease	1	2773	1360 (49.0)	
Diabetes mellitus	4	30259	13923 (46.0)	
Hypercholesterolemia	4	37103	17233 (46.4)	
Hypercholesterolemia with risk factors for ASCVD	2	25210	13184 (52.3)	
Risk factors for ASCVD without hypercholesterolemia	1	25871	16023 (61.9)	
Acute coronary syndrome	5	54515	19211 (35.2)	
Stable coronary heart disease	6	87897	42303 (48.1)	

© 2020 Khan SU et al. *JAMA Network Open.*

Heart failure	0	0	0	
**Location**				
North America	8	71161	35727 (50.2)	0.82
Western Europe	3	37312	18648 (50.0)	
Multiregional	10	136346	61393 (45.0)	
Rest of the world	2	18809	7469 (39.7)	
**Funding**				
Industry	12	123940	51863 (41.8)	0.32
Government	2	31389	17881 (57.0)	
University/organization	2	14066	5367 (38.2)	
Other/combined	7	94233	48126 (51.1)	

ASCVD, Atherosclerotic Cardiovascular Disease; NR, Not Reported

© 2020 Khan SU et al. *JAMA Network Open.*

eTable 8. Trends in Randomized Clinical Trials Reporting Outcomes Based on Sex and Age

Characteristics	1990-1994	1995-1998	1999-2002	2003-2006	2007-2010	2011-2014	2015-2018	P-value
Trials reporting outcomes in women, No. (%)	NR	3 (60)	5 (45.5)	5 (38.5)	7 (58.3)	4 (66.7)	8 (72.7)	0.42
Women participants, No. (%)	NR	3095 (15.6)	14064 (33)	12985 (35.5)	26617 (39.9)	16105 (31.7)	43943 (34)	0.39
Trials reporting outcomes in participants ≥ 65 yrs., No. (%)	NR	1 (25)	5 (50)	3 (25)	3 (30)	4 (80)	6 (60)	0.20
Participants age ≥ 65 yrs., No. (%)	NR	3514 (39)	19082 (48.6)	5185 (28.1)	12479 (47.8)	28102 (51.9)	36566 (41.6)	0.81
**Women**								
**Therapy, No (%)**								
Statins	NR	3 (17.6)	5 (29.4)	4 (23.5)	4 (23.5)	NR	1(5.9)	0.01
Ezetimibe	NR	NR	NR	NR	NR	1 (50)	1 (50)	
PCSK9 inhibitors	NR	NR	NR	NR	NR	NR	3 (100)	
Fibrates	NR	NR	NR	1 (50)	1 (50)	NR	NR	
Niacin	NR	NR	NR	NR	NR	2 (100)	NR	
Omega 3 fatty acids	NR	NR	NR	NR	2 (33.3)	1 (16.7)	3 (50)	
**Indication or baseline population, No. (%)**								0.82
Aortic stenosis	NR	NR	NR	NR	NR	NR	NR	
Chronic kidney disease	NR	NR	NR	NR	1 (50)	1 (50)	NR	
Diabetes mellitus	NR	NR	NR	1 (33.3)	1 (33.3)	NR	1 (33.3)	
Hypercholesterolemia	NR	1 (16.7)	1 (16.7)	1 (16.7)	2 (33.3)	NR	1 (16.7)	
Hypercholesterolemia with risk factors for ASCVD	NR	NR	1 (33.3)	NR	NR	1 (33.3)	1 (33.3)	
Risk factors for ASCVD without hypercholesterolemia	NR	NR	NR	1 (50)	NR	NR	1 (50)	
Acute coronary syndrome	NR	2 (22.2)	1 (11.2)	2 (22.2)	2 (22.2)	NR	2 (22.2)	
Stable coronary heart disease	NR	NR	2 (33.3)	NR	NR	2 (33.3)	2 (33.3)	
Heart failure	NR	NR	NR	NR	1 (100)	NR	NR	
**Setting, No. (%)**								
Primary prevention	NR	1 (6.3)	2 (12.5)	3 (18.8)	4 (25)	2 (12.5)	4 (25)	0.97

© 2020 Khan SU et al. *JAMA Network Open.*

Secondary prevention	NR	2 (12.5)	3 (18.8)	2 (12.5)	3 (18.8)	2 (12.5)	4 (25)	
**Location, No. (%)**								
North America	NR	2 (25)	1 (12.5)	1 (12.5)	1 (12.5)	1 (12.5)	2 (25)	0.15
Western Europe	NR	NR	4 (40)	1 (10)	2 (20)	2 (20)	1 (10)	
Multiregional	NR	NR	NR	1 (10)	3 (30)	1 (10)	5 (50)	
Rest of the world	NR	1 (25)	NR	2 (50)	1 (25)	NR	NR	
**Funding, No (%)**								
Industry	NR	1 (6.7)	1 (6.7)	3 (20)	4 (26.7)	1 (6.7)	5 (33.3)	0.55
Government	NR	NR	NR	NR	1 (50)	NR	1 (50)	
University/organization	NR	NR	2 (50)	1 (25)	1 (25)	NR	NR	
Other/combined	NR	2 (18.2)	2 (18.2)	1 (9.1)	1 (9.1)	3 (27.3)	2 (18.2)	
**Age ≥ 65 years**								
**Therapy, No (%)**								
Statins	NR	1 (11.1)	3 (33.3)	2 (22.2)	2 (22.2)	NR	1 (11.1)	0.11
Ezetimibe	NR	NR	NR	NR	NR	NR	1 (100)	
PCSK9 inhibitors	NR	NR	NR	NR	NR	NR	3 (100)	
Fibrates	NR	NR	2 (50)	1 (25)	1 (25)	NR	NR	
Niacin	NR	NR	NR	NR	NR	2 (100)	NR	
Omega 3 fatty acids	NR	NR	NR	NR	NR	2 (66.6)	1 (33.3)	
**Indication or baseline population, No. (%)**								
Aortic stenosis	NR	NR	NR	NR	NR	NR	NR	0.50
Chronic kidney disease	NR	NR	NR	NR	1 (100)	NR	NR	
Diabetes mellitus	NR	NR	NR	1 (33.3)	1 (33.3)	1 (33.3)	NR	
Hypercholesterolemia	NR	NR	1 (33.3)	NR	1 (33.3)	NR	1 (33.3)	
Hypercholesterolemia with risk factors for ASCVD	NR	NR	NR	NR	NR	1 (50)	1 (50)	
Risk factors for ASCVD without hypercholesterolemia	NR	NR	NR	NR	NR	NR	NR	
Acute coronary syndrome	NR	1 (16.7)	1 (16.7)	2 (33.3)	NR	NR	2 (33.3)	
Stable coronary heart disease	NR	NR	3 (42.9)	NR	NR	2 (28.6)	2 (28.6)	
Heart failure	NR	NR	NR	NR	NR	NR	NR	
**Setting, No. (%)**								

© 2020 Khan SU et al. *JAMA Network Open.*

Primary prevention	NR	NR	1 (11.1)	1 (11.1)	3 (33.3)	2 (22.2)	2 (22.2)	0.28
Secondary prevention	NR	1 (7.7)	4 (30.8)	2 (15.4)	NR	2 (15.4)	4 (30.8)	
**Location, No. (%)**								
North America	NR	NR	2 (33.3)	1 (16.7)	1 (16.7)	1 (16.7)	1 (16.7)	0.04
Western Europe	NR	NR	3 (75)	NR	NR	1 (25)	NR	
Multiregional	NR	NR	NR	1 (10)	2 (20)	2 (20)	5 (50)	
Rest of the world	NR	1 (50)	NR	1 (50)	NR	NR	NR	
**Funding, No (%)**								
Industry	NR	NR	1 (9.1)	2 (18.2)	2 (18.2)	1 (9.1)	5 (45.5)	0.12
Government	NR	NR	NR	NR	1(100)	NR	NR	
University/organization	NR	NR	2 (66.6)	1 (33.3)	NR	NR	NR	
Other/combined	NR	1 (14.3)	2 (28.6)	NR	NR	3 (42.9)	1 (14.3)	

ASCVD, Atherosclerotic Cardiovascular Disease; NR, Not Reported; NA, Not Applicable

© 2020 Khan SU et al. *JAMA Network Open.*

eReferences

1. Effects of pravastatin in patients with serum total cholesterol levels from 5.2 to 7.8 mmol/liter (200 to 300 mg/dl) plus two additional atherosclerotic risk factors. The Pravastatin Multinational Study Group for Cardiac Risk Patients. *The American journal of cardiology.* 1993;72(14):1031-1037.
2. Randomised trial of cholesterol lowering in 4444 patients with coronary heart disease: the Scandinavian Simvastatin Survival Study (4S). *Lancet.* 1994;344(8934):1383-1389.
3. Shepherd J, Cobbe SM, Ford I, et al. Prevention of coronary heart disease with pravastatin in men with hypercholesterolemia. West of Scotland Coronary Prevention Study Group. *N Engl J Med.* 1995;333(20):1301-1307.
4. Sacks FM, Pfeffer MA, Moye LA, et al. The effect of pravastatin on coronary events after myocardial infarction in patients with average cholesterol levels. Cholesterol and Recurrent Events Trial investigators. *N Engl J Med.* 1996;335(14):1001-1009.
5. The effect of aggressive lowering of low-density lipoprotein cholesterol levels and low-dose anticoagulation on obstructive changes in saphenous-vein coronary-artery bypass grafts. *The New England journal of medicine.* 1997;336(3):153-162.
6. Downs JR, Clearfield M, Weis S, et al. Primary prevention of acute coronary events with lovastatin in men and women with average cholesterol levels: results of AFCAPS/TexCAPS. Air Force/Texas Coronary Atherosclerosis Prevention Study. *Jama.* 1998;279(20):1615-1622.
7. Prevention of cardiovascular events and death with pravastatin in patients with coronary heart disease and a broad range of initial cholesterol levels. *The New England journal of medicine.* 1998;339(19):1349-1357.
8. Results of the low-dose (20 mg) pravastatin GISSI Prevenzione trial in 4271 patients with recent myocardial infarction: do stopped trials contribute to overall knowledge? GISSI Prevenzione Investigators (Gruppo Italiano per lo Studio della Sopravvivenza nell'Infarto Miocardico). *Italian heart journal : official journal of the Italian Federation of Cardiology.* 2000;1(12):810-820.
9. Schwartz GG, Olsson AG, Ezekowitz MD, et al. Effects of atorvastatin on early recurrent ischemic events in acute coronary syndromes: the MIRACL study: a randomized controlled trial. *Jama.* 2001;285(13):1711-1718.
10. Major outcomes in moderately hypercholesterolemic, hypertensive patients randomized to pravastatin vs usual care: The Antihypertensive and Lipid-Lowering Treatment to Prevent Heart Attack Trial (ALLHAT-LLT). *Jama.* 2002;288(23):2998-3007.
11. Athyros VG, Papageorgiou AA, Mercouris BR, et al. Treatment with atorvastatin to the National Cholesterol Educational Program goal versus 'usual' care in secondary coronary heart disease prevention. The GREek Atorvastatin and Coronary-heart-disease Evaluation (GREACE) study. *Current medical research and opinion.* 2002;18(4):220-228.
12. MRC/BHF Heart Protection Study of cholesterol lowering with simvastatin in 20,536 high-risk individuals: a randomised placebo-controlled trial. *Lancet.* 2002;360(9326):7-22.
13. Serruys PW, de Feyter P, Macaya C, et al. Fluvastatin for prevention of cardiac events following successful first percutaneous coronary intervention: a randomized controlled trial. *Jama.* 2002;287(24):3215-3222.
14. Shepherd J, Blauw GJ, Murphy MB, et al. Pravastatin in elderly individuals at risk of vascular disease (PROSPER): a randomised controlled trial. *Lancet.* 2002;360(9346):1623-1630.
15. Holdaas H, Fellstrom B, Jardine AG, et al. Effect of fluvastatin on cardiac outcomes in renal transplant recipients: a multicentre, randomised, placebo-controlled trial. *Lancet.* 2003;361(9374):2024-2031.

© 2020 Khan SU et al. *JAMA Network Open.*

16. Sever PS, Dahlof B, Poulter NR, et al. Prevention of coronary and stroke events with atorvastatin in hypertensive patients who have average or lower-than-average cholesterol concentrations, in the Anglo-Scandinavian Cardiac Outcomes Trial--Lipid Lowering Arm (ASCOT-LLA): a multicentre randomised controlled trial. *Lancet.* 2003;361(9364):1149-1158.
17. de Lemos JA, Blazing MA, Wiviott SD, et al. Early intensive vs a delayed conservative simvastatin strategy in patients with acute coronary syndromes: phase Z of the A to Z trial. *Jama.* 2004;292(11):1307-1316.
18. Koren MJ, Hunninghake DB. Clinical outcomes in managed-care patients with coronary heart disease treated aggressively in lipid-lowering disease management clinics: the alliance study. *Journal of the American College of Cardiology.* 2004;44(9):1772-1779.
19. Colhoun HM, Betteridge DJ, Durrington PN, et al. Primary prevention of cardiovascular disease with atorvastatin in type 2 diabetes in the Collaborative Atorvastatin Diabetes Study (CARDS): multicentre randomised placebo-controlled trial. *Lancet.* 2004;364(9435):685-696.
20. Cannon CP, Braunwald E, McCabe CH, et al. Intensive versus moderate lipid lowering with statins after acute coronary syndromes. *N Engl J Med.* 2004;350(15):1495-1504.
21. LaRosa JC, Grundy SM, Waters DD, et al. Intensive lipid lowering with atorvastatin in patients with stable coronary disease. *N Engl J Med.* 2005;352(14):1425-1435.
22. Wanner C, Krane V, Marz W, et al. Atorvastatin in patients with type 2 diabetes mellitus undergoing hemodialysis. *The New England journal of medicine.* 2005;353(3):238-248.
23. Pedersen TR, Faergeman O, Kastelein JJ, et al. High-dose atorvastatin vs usual-dose simvastatin for secondary prevention after myocardial infarction: the IDEAL study: a randomized controlled trial. *Jama.* 2005;294(19):2437-2445.
24. Knopp RH, d'Emden M, Smilde JG, Pocock SJ. Efficacy and safety of atorvastatin in the prevention of cardiovascular end points in subjects with type 2 diabetes: the Atorvastatin Study for Prevention of Coronary Heart Disease Endpoints in non-insulin-dependent diabetes mellitus (ASPEN). *Diabetes care.* 2006;29(7):1478-1485.
25. Nakamura H, Arakawa K, Itakura H, et al. Primary prevention of cardiovascular disease with pravastatin in Japan (MEGA Study): a prospective randomised controlled trial. *Lancet.* 2006;368(9542):1155-1163.
26. Amarenco P, Bogousslavsky J, Callahan A, 3rd, et al. High-dose atorvastatin after stroke or transient ischemic attack. *N Engl J Med.* 2006;355(6):549-559.
27. Kjekshus J, Apetrei E, Barrios V, et al. Rosuvastatin in older patients with systolic heart failure. *The New England journal of medicine.* 2007;357(22):2248-2261.
28. Ridker PM, Danielson E, Fonseca FA, et al. Rosuvastatin to prevent vascular events in men and women with elevated C-reactive protein. *N Engl J Med.* 2008;359(21):2195-2207.
29. Tavazzi L, Maggioni AP, Marchioli R, et al. Effect of rosuvastatin in patients with chronic heart failure (the GISSI-HF trial): a randomised, double-blind, placebo-controlled trial. *Lancet (London, England).* 2008;372(9645):1231-1239.
30. Fellstrom BC, Jardine AG, Schmieder RE, et al. Rosuvastatin and cardiovascular events in patients undergoing hemodialysis. *The New England journal of medicine.* 2009;360(14):1395-1407.
31. Armitage J, Bowman L, Wallendszus K, et al. Intensive lowering of LDL cholesterol with 80 mg versus 20 mg simvastatin daily in 12,064 survivors of myocardial infarction: a double-blind randomised trial. *Lancet.* 2010;376(9753):1658-1669.
32. Yusuf S, Bosch J, Dagenais G, et al. Cholesterol Lowering in Intermediate-Risk Persons without Cardiovascular Disease. *N Engl J Med.* 2016;374(21):2021-2031.
33. Rossebo AB, Pedersen TR, Boman K, et al. Intensive lipid lowering with simvastatin and ezetimibe in aortic stenosis. *N Engl J Med.* 2008;359(13):1343-1356.

© 2020 Khan SU et al. *JAMA Network Open.*

34. Baigent C, Landray MJ, Reith C, et al. The effects of lowering LDL cholesterol with simvastatin plus ezetimibe in patients with chronic kidney disease (Study of Heart and Renal Protection): a randomised placebo-controlled trial. *Lancet.* 2011;377(9784):2181-2192.
35. Cannon CP, Blazing MA, Giugliano RP, et al. Ezetimibe Added to Statin Therapy after Acute Coronary Syndromes. *N Engl J Med.* 2015;372(25):2387-2397.
36. Robinson JG, Farnier M, Krempf M, et al. Efficacy and safety of alirocumab in reducing lipids and cardiovascular events. *The New England journal of medicine.* 2015;372(16):1489-1499.
37. Sabatine MS, Giugliano RP, Wiviott SD, et al. Efficacy and safety of evolocumab in reducing lipids and cardiovascular events. *N Engl J Med.* 2015;372(16):1500-1509.
38. Sabatine MS, Giugliano RP, Keech AC, et al. Evolocumab and Clinical Outcomes in Patients with Cardiovascular Disease. *N Engl J Med.* 2017;376(18):1713-1722.
39. Ridker PM, Revkin J, Amarenco P, et al. Cardiovascular Efficacy and Safety of Bococizumab in High-Risk Patients. *The New England journal of medicine.* 2017;376(16):1527-1539.
40. Schwartz GG, Steg PG, Szarek M, Bhatt DL. Alirocumab and Cardiovascular Outcomes after Acute Coronary Syndrome. 2018;379(22):2097-2107.
41. Secondary prevention by raising HDL cholesterol and reducing triglycerides in patients with coronary artery disease. *Circulation.* 2000;102(1):21-27.
42. Meade T, Zuhrie R, Cook C, Cooper J. Bezafibrate in men with lower extremity arterial disease: randomised controlled trial. *Bmj.* 2002;325(7373):1139.
43. Keech A, Simes RJ, Barter P, et al. Effects of long-term fenofibrate therapy on cardiovascular events in 9795 people with type 2 diabetes mellitus (the FIELD study): randomised controlled trial. *Lancet (London, England).* 2005;366(9500):1849-1861.
44. Ginsberg HN, Elam MB, Lovato LC, et al. Effects of combination lipid therapy in type 2 diabetes mellitus. *The New England journal of medicine.* 2010;362(17):1563-1574.
45. Rubins HB, Robins SJ, Collins D, et al. Gemfibrozil for the secondary prevention of coronary heart disease in men with low levels of high-density lipoprotein cholesterol. Veterans Affairs High-Density Lipoprotein Cholesterol Intervention Trial Study Group. *The New England journal of medicine.* 1999;341(6):410-418.
46. Boden WE, Probstfield JL, Anderson T, et al. Niacin in patients with low HDL cholesterol levels receiving intensive statin therapy. *The New England journal of medicine.* 2011;365(24):2255-2267.
47. Landray MJ, Haynes R, Hopewell JC, et al. Effects of extended-release niacin with laropiprant in high-risk patients. *The New England journal of medicine.* 2014;371(3):203-212.
48. Bonds DE, Harrington M, Worrall BB, et al. Effect of long-chain omega-3 fatty acids and lutein + zeaxanthin supplements on cardiovascular outcomes: results of the Age-Related Eye Disease Study 2 (AREDS2) randomized clinical trial. *JAMA internal medicine.* 2014;174(5):763-771.
49. Galan P, Kesse-Guyot E, Czernichow S, Briancon S, Blacher J, Hercberg S. Effects of B vitamins and omega 3 fatty acids on cardiovascular diseases: a randomised placebo controlled trial. *Bmj.* 2010;341:c6273.
50. Yokoyama M, Origasa H, Matsuzaki M, et al. Effects of eicosapentaenoic acid on major coronary events in hypercholesterolaemic patients (JELIS): a randomised open-label, blinded endpoint analysis. *Lancet (London, England).* 2007;369(9567):1090-1098.
51. Kromhout D, Giltay EJ, Geleijnse JM. n-3 fatty acids and cardiovascular events after myocardial infarction. *The New England journal of medicine.* 2010;363(21):2015-2026.
52. Rauch B, Schiele R, Schneider S, et al. OMEGA, a randomized, placebo-controlled trial to test the effect of highly purified omega-3 fatty acids on top of modern guideline-adjusted therapy after myocardial infarction. *Circulation.* 2010;122(21):2152-2159.

© 2020 Khan SU et al. *JAMA Network Open.*

53. Roncaglioni MC, Tombesi M, Avanzini F, et al. n-3 fatty acids in patients with multiple cardiovascular risk factors. *The New England journal of medicine.* 2013;368(19):1800-1808.
54. Tavazzi L, Maggioni AP, Marchioli R, et al. Effect of n-3 polyunsaturated fatty acids in patients with chronic heart failure (the GISSI-HF trial): a randomised, double-blind, placebo-controlled trial. *Lancet (London, England).* 2008;372(9645):1223-1230.
55. Bosch J, Gerstein HC, Dagenais GR, et al. n-3 fatty acids and cardiovascular outcomes in patients with dysglycemia. *The New England journal of medicine.* 2012;367(4):309-318.
56. Dietary supplementation with n-3 polyunsaturated fatty acids and vitamin E after myocardial infarction: results of the GISSI-Prevenzione trial. Gruppo Italiano per lo Studio della Sopravvivenza nell'Infarto miocardico. *Lancet (London, England).* 1999;354(9177):447-455.
57. Manson JE, Cook NR, Lee IM, et al. Marine n-3 Fatty Acids and Prevention of Cardiovascular Disease and Cancer. *The New England journal of medicine.* 2019;380(1):23-32.
58. Bowman L, Mafham M, Wallendszus K, et al. Effects of n-3 Fatty Acid Supplements in Diabetes Mellitus. *The New England journal of medicine.* 2018;379(16):1540-1550.
59. Bhatt DL, Steg PG, Miller M, et al. Cardiovascular Risk Reduction with Icosapent Ethyl for Hypertriglyceridemia. *N Engl J Med.* 2019;380(1):11-22.

© 2020 Khan SU et al. *JAMA Network Open.*
